# Acceleration of sequence clustering using longest common subsequence filtering

**DOI:** 10.1186/1471-2105-14-S8-S7

**Published:** 2013-05-09

**Authors:** Youhei Namiki, Takashi Ishida, Yutaka Akiyama

**Affiliations:** 1 Department of Computer Science, Graduate School of Information Science and Engineering, Tokyo Institute of Technology, Ookayama, Meguro, Tokyo 152-8552, Japan

## Abstract

**Background:**

Huge numbers of genomes can now be sequenced rapidly with recent improvements in sequencing throughput. However, data analysis methods have not kept up, making it difficult to process the vast amounts of available sequence data. This increased processing time is especially critical in DNA sequence clustering because of the intrinsic difficulty in parallelization. Thus, there is a strong demand for a faster clustering algorithm.

**Results:**

We developed a new fast DNA sequence clustering method called LCS-HIT, based on the popular CD-HIT program. The proposed method uses a novel filtering technique based on the longest common subsequence to identify similar sequence pairs. This filtering technique makes the LCS-HIT considerably faster than CD-HIT, without loss of sensitivity. For a dataset of two million DNA sequences, our method was approximately 7.1, 4.4, and 2.2 times faster than CD-HIT for 100, 150, and 400 bases, respectively.

**Conclusions:**

The LCS-HIT clustering program, using a novel filtering technique based on the longest common subsequence, is significantly faster than CD-HIT without compromising clustering accuracy. Moreover, the filtering technique itself is independent from the CD-HIT algorithm. Thus, this technique can be applied to similar clustering algorithms.

## Background

Clustering is a data mining method that aims to identify similar groups in huge datasets, and is widely used in various bioinformatics fields, such as cancer class discovery [1] and protein structure prediction [2]. Biological sequence clustering is one of the main applications of clustering in bioinformatics, and has two main objectives: first, to reduce the size of the dataset by identifying representatives for each cluster and removing redundant sequences; and second, to find sequence patterns that appear in the dataset by checking cluster sizes (the number of members).

Recent progress in DNA sequencing has enabled us to amass huge amounts of genomic data in a short time. While current sequencers produce relatively short sequences (≈150 bases), the number of such sequences is huge (≈10 million). However, the analyses of these vast amounts of data require a considerable amount of time. This necessitates a shift in focus from sequencing throughput to the computational speed of algorithms for sequence data analysis. Generally, clustering algorithms require *O*(*N*^2^) time for *N *sequences, making it difficult to scale them to large datasets. Several algorithms have been developed for more efficient sequence clustering for very similar sequences [3]; however, it is still difficult to rapidly group remote homologous sequences into a cluster based on sequence identity [4,5].

For clustering based on sequence identity, CD-HIT [6,7] is one of the most widely used sequence clustering tools. CD-HIT is used on public databases such as Uniprot [8] and PDB [9] to remove redundant sequences, and has also been used for various bioinformatics analysis, including metagenomic data analysis [10]. It clusters sequences on the basis of sequence identity between pairs, and can deal with large datasets in a relatively short time by relying on an approximate clustering approach and short word filtering. Short word filtering greatly decreases the burden of the sequence alignment calculation by identifying similar sequences on the basis of matches between short subsequences. However, although this filtering scheme is very computationally efficient, it is too approximate to exclude many dissimilar sequence pairs. Thus, even this well-known tool requires at least 2 days to cluster 10 million sequences. Furthermore, given the potential of the next generation of sequencers, it is clear that further speed enhancement is necessary.

Here, we present a faster and more accurate clustering program named LCS-HIT. We introduce a novel filtering technique to select similar sequence pairs on the basis of the longest common subsequence (LCS) before the sequence alignment process. This approach is much faster than sequence alignment and is stricter than short word filtering. Thus, our clustering algorithm is significantly faster than CD-HIT without compromising on clustering accuracy.

The program and source code of the LCS-HIT, which are freely available for download at http://www.bi.cs.titech.ac.jp/lcshit/, are implemented in C++ and are supported on Linux with GCC (version 4 or later) and GNU make.

## Methods

### Algorithm

**Figure 1:**
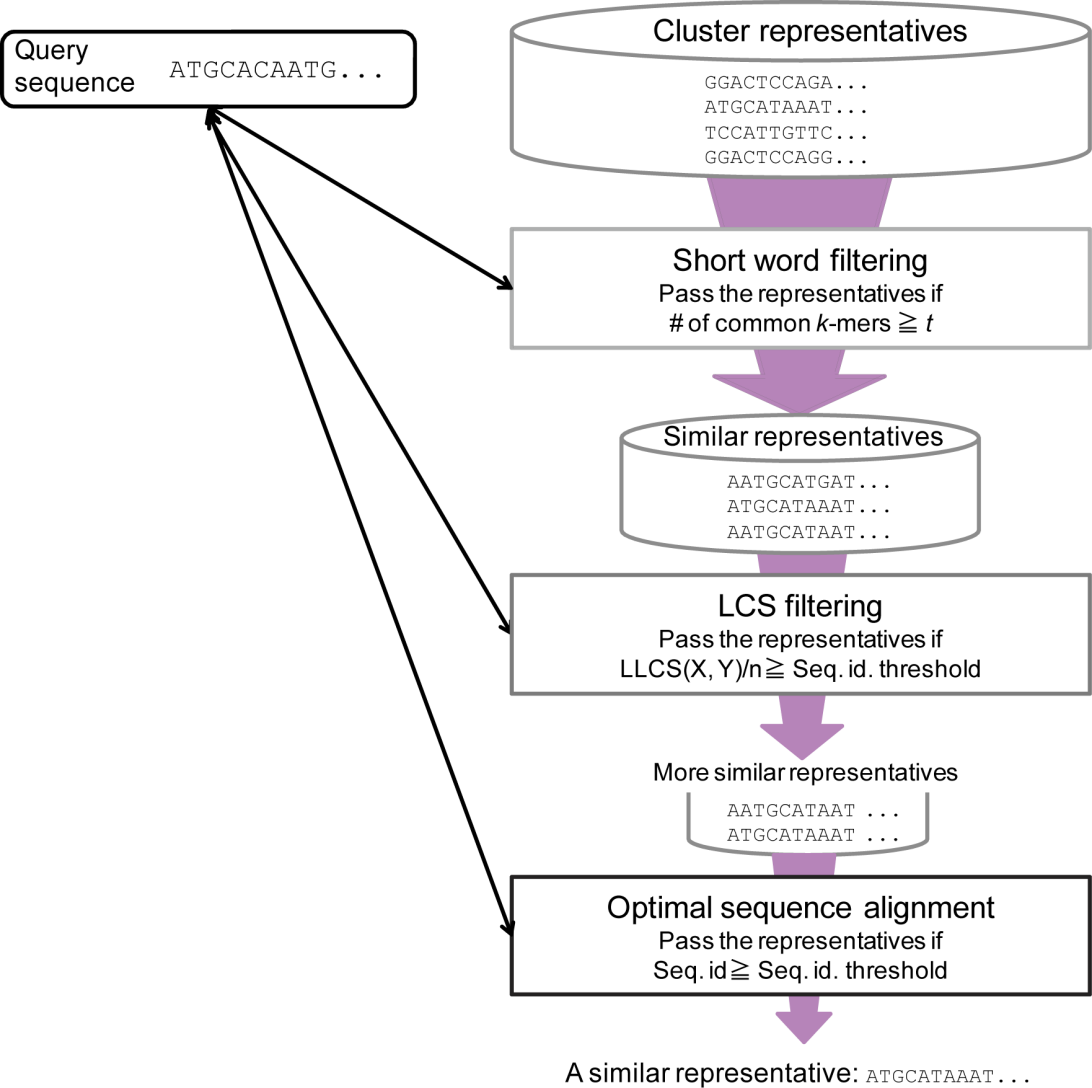
**Flowchart of proposed method**.

Our sequence clustering method consists of several parts, as shown in Figure 1, and operates according to the following sequence of steps:

Let *Q *be the set of sequences to be clustered and *R*=∅ be the set of existing cluster representatives.

For each q∈Q,

(a) Compare *q *and *R *by using the short word table, and find the set $R_{q,k}\left(\subseteq R\right)$, the subset of representatives with ≥t common *k*-mers (partial sequences of *k *nucleotides) with *q ***(short word filtering)**.

(b) For each *q *and each $r\in R_{q,k}$,

(i) Compute the length of the longest common subsequence *LLCS*(*q, r*) between *q *and *r*. If *LLCS*(*q, r*) is above a certain threshold, save the pair as a candidate similar sequence pair that may belong to the same cluster. Otherwise, *q *and *r *are considered to belong to different clusters **(LCS filtering)**.

(ii) For each saved sequence pair *q *and *r*, compute the optimal sequence alignment using affine gap penalties and the sequence identity between *q *and *r*. If the sequence identity is greater than or equal to the threshold *s*, then *q *and *r *are considered to belong to the same cluster, and *q *is added to *r*'s cluster (the cluster representative is not updated). Otherwise, they are considered to belong to different clusters.

(c) If *q *does not belong to any existing cluster, then create a new cluster whose member and representative is *q. q *is also registered in the short word table, and let R←R∪q.

An optimal alignment must be computed when checking whether a pair of sequences belongs to the same cluster. However, the time complexity of computing an optimal alignment is *O*(*mn*) by dynamic programming, where *m *and *n *are the lengths of the two sequences. This is very slow, making it unrealistic to attempt to compute alignments for all combinations of sequences. Additionally, the number of sequence pairs with a sequence identity less than the threshold *s *is much more than the number of similar sequence pairs with a sequence identity above the threshold. Thus, our method filters similar sequence pairs by a faster method in advance, and prunes the comparison set of dissimilar pairs. As a result, the number of alignment computations dramatically decreases, and the whole clustering process can be completed in a reasonable length of time. In the following sections, we describe the two filtering methods: short word filtering and the proposed LCS filtering. When a new cluster is created, the first assigned sequence is selected as the representative for its cluster. Representatives of existing clusters are not updated, even if new sequences are added to the clusters. This means that the clustering result depends on the order of input sequences, while the computational cost in comparing an input sequence with the cluster representative can be significantly reduced. Our clustering method is largely based on CD-HIT and retains many similarities. However, there are two main differences; our method employs a new filtering process (LCS filtering) after short word filtering, and different filtering criteria are used for short word filtering.

### Filtering similar sequence pairs based on the longest common subsequence

Short word filtering can process large amounts of sequence data in a short time because of the low computational complexity. Thus, it is suitable for filtering large sequence datasets. However, the filtering is rough: many dissimilar sequence pairs, i.e., with sequence identity less than the threshold s, pass through the short word filter. Thus, the subsequent sequence alignment process will be hampered and the overall computation time for clustering will be prolonged. Instead, it is necessary to introduce a fast and accurate filtering process after the rough, short word filtering stage. Here, we introduce a new filtering method that relies on the relationship between the length of the LCS and the sequence identity. Although the computational cost for determining the LCS is generally not negligible, a bit-parallel algorithm can be used to accelerate its calculation and avoid a bottleneck.

### The longest common subsequence

Let a subsequence be created by picking up elements from a main sequence while preserving their relative order. The LCS is the longest common subsequence among all possible common subsequences. For example, "TAGC" is the LCS of "ATCAGTC" and "CTAGAC." Finding the LCS of a sequence pair is equivalent to aligning the two sequences to maximize the number of the matched elements.

### The length of the longest common subsequence

The LCS of two sequences $X=\left(x_{1},x_{2},\ldots ,x_{m}\right)$ and $Y=\left(y_{1},y_{2},\ldots ,y_{n}\right)$ can be computed as follows:

Let $X_{i}=\left(x_{1},x_{2},\ldots ,x_{i}\right)\left(0\leq i\leq m\right)$ and $Y_{j}=\left(y_{1},y_{2},\ldots ,y_{j}\right)\left(0\leq j\leq n\right)$; and let *LLCS*(*X, Y*) be the length of the LCS between *X *and *Y*. Then,

$$LLCS\left(X_{i},Y_{j}\right)=\left\{\begin{array}{ccc} 0 & & \left(\text{if }i=0\text{ or }j=0\right)\\ LLCS\left(X_{i-1},Y_{j-1}\right)+1 & & \left(\text{if }x_{i}=y_{j}\right)\\ \text{max}\left(LLCS\left(X_{i},Y_{j-1}\right),LLCS\left(X_{i-1},Y_{j}\right)\right) & & \left(\text{if }x_{i}\neq y_{j}\right) \end{array}\right)$$

Generally, *LLCS*(*X, Y*) can be computed by dynamic programming, which has an *O*(*mn*) time complexity.

### LCS filtering for similar sequence pairs

The length of the LCS of a sequence pair, *LLCS*(*X, Y*), equals the number of matches in the sequence alignment that maximizes matches between the two sequences. Suppose the sequence *X *is longer than or of equal length to Y(m≥n). The sequence identity of a sequence pair equals the number of matches in the alignment that maximizes the alignment score for the two sequences divided by the shorter sequence length *n*.

From this, the following relationship holds between the length of the LCS and the sequence identity.

(1) $$\frac{LLCS\left(X,Y\right)}{n}\geq \text{Sequence identity}$$

This means that the ratio of *LLCS*(*X, Y*) to the sequence length *n *equals the upper boundary of the sequence identity of the two sequences. Using this relationship, dissimilar sequence pairs whose sequence identity is less than the threshold *s *can be pruned by checking whether $LLCS\left(X,Y\right)/n)$ is greater than *s *before computing the sequence identity.

### Advantages of LCS filtering

First, LCS filtering can be performed with the threshold of sequence identity *s *as the filtering criterion and does not need new heuristic thresholds. Second, sequence pairs with sequence identity greater than the given threshold *s *always pass LCS filtering (no false negatives), because $LLCS\left(X,Y\right)/n)$ is always larger than the sequence identity. Thus, LCS filtering can be considered a suitable filtering process to follow the short word filter and precede the alignment computation.

### Fast bit-parallel LLCS computation

As described above, using *LLCS*(*X*,*Y*), we can filter similar sequence pairs more accurately than the short word filter. However, the time complexity of the *LLCS*(*X*,*Y*) computation by dynamic programming is *O*(*mn*), which is too large.

Nevertheless, there are several bit-parallel LCS-length computation algorithms whose time complexity is almost *O*(*n*), which can compute *LLCS*(*X*,*Y*) in a reasonable amount of time [11-13]. We used the most efficient of Hyyro's bit-parallel algorithms to reduce the computation time for *LLCS*(*X*,*Y*).

Here, $b^{i}$ denotes *i *repetitions of bit *b*. With this notation, we can write $1111=1^{4}$ and $0011100=0^{2}1^{3}0^{2}$. Additionally, let Σ  be the set of alphabets that appear in *X *and *Y*, and let $\sigma =\left|\Sigma \right|$ be the number of alphabets.

**Figure 2:**
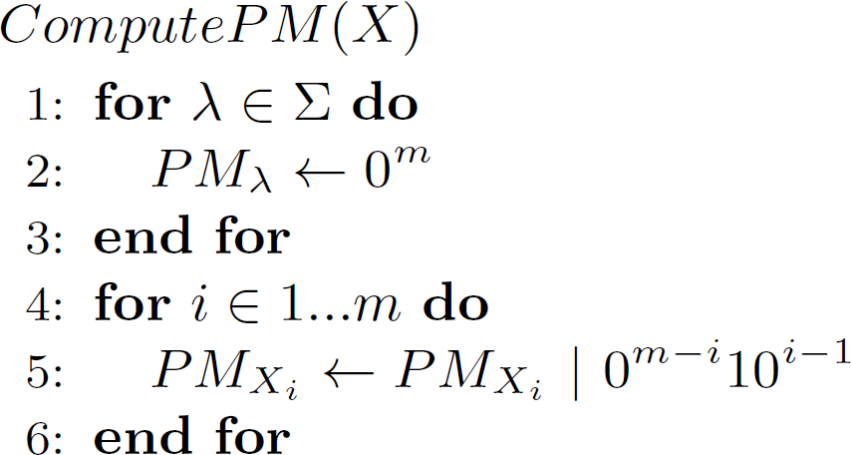
**Computation of *ComputePM*(*X*)**.

**Figure 3:**
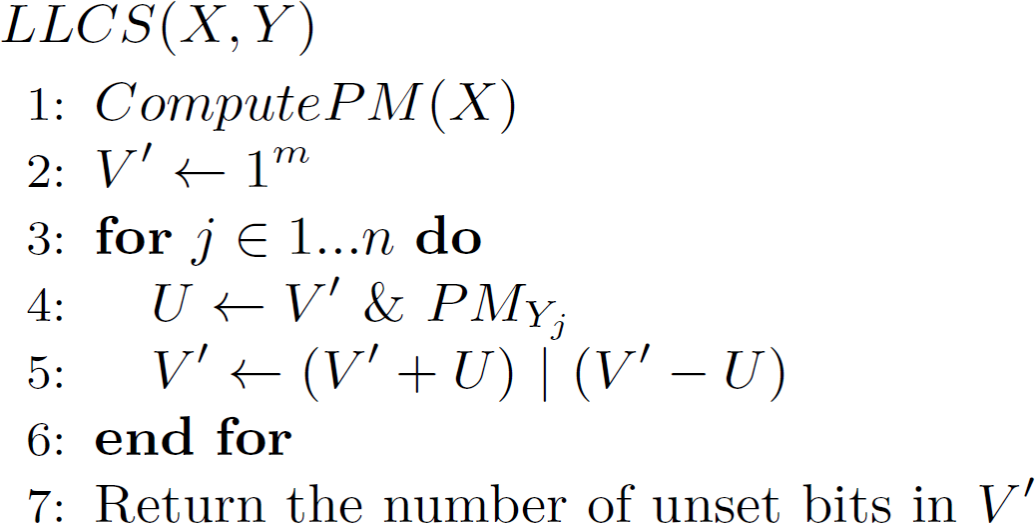
**Computation of *LLCS*(*X, Y*) by bit-parallel algorithm**.

Let *ComputePM*(*X*) be defined as in Figure 2, where "|" denotes the bitwise OR operation. *ComputePM*(*X*) sets the corresponding positions of bits of *PM *(position matrix) for each nucleotide in the sequence *X*. With this *ComputePM*(*X*), *LLCS*(*X, Y*) can be computed as shown in Figure 3. In the figure, "&" denotes the bitwise AND operation, and + and - denote arithmetic addition and subtraction of integers, respectively. These operations will require carries and borrows between adjacent bits.

The time complexity of *ComputePM*(*X*) is, $O\left(\sigma \left\lceil m/w)\right\rceil +m\right)$ and that of *LLCS *is $O\left(\sigma \left\lceil m/w)\right\rceil n\right)$, where w is the bit-length of a variable used for storing sequence data. Therefore, we can see that the computation of *ComputePM*(*X*) and *LLCS*(*X*, *Y*) is much faster than dynamic programming, and also faster than the alignment computation.

### Modification of short word filtering

Short word filtering is a fast filtering method for finding similar sequence pairs between existing cluster representatives and a query sequence. It checks for the number of common *k*-mers (partial sequences of *k *nucleotides) between the two to find a match. The theory behind this method is that a pair of similar sequences with high sequence identity must have short identical words. The value *k *should be decided according to the length of the query sequences and the threshold of sequence identity. The short word filter is used in CD-HIT, but in the proposed method, we use different filtering criteria to accommodate the subsequent LCS filter. In short word filtering, *k*-mers in the representatives are indexed in an index table (short word table) in advance; thus, cluster representatives with specific *k*-mers can be filtered quickly.

### Registering cluster representatives with the short word table

When a new cluster is created, its representative must be registered with the short word table. First, every *k*th *k*-mer of a new cluster representative sequence is enumerated. Next, all the enumerated *k*-mers are indexed in the short word table and associated with the representative of the cluster that the *k*-mers belong to.

The short word table is implemented in the form of an index table. This data structure enables rapid enumeration of cluster representatives that contain a specified *k*-mer. In the case of nucleotides, the number of entries in the index table should be at least 4*^k ^*since there are four DNA bases and the number of possible *k*-mers is 4*^k^*.

### Searching cluster representatives in the short word table

When searching for cluster representatives that have common *k*-mers with the query sequence, all *k*-mers in the query sequence must be first enumerated. Then, cluster representatives with those *k*-mers are searched using the short word table. If there are *t *or more common *k*-mers between the query and a cluster representative, this pair of sequences passes the short word filter as a candidate of similar sequences belonging to the same cluster. Dissimilar sequence pairs, i.e., those with fewer than *t *common *k*-mers, are discarded.

The threshold *t *should be set according to the length of sequences, *k*, and the threshold of the sequence identity *s*. In our method, *t *is set relatively low because our short word filtering only enumerates every *k*th *k*-mer of the cluster representatives. Ideally, *t *should be $t^{\prime }/k)$, where $t^{\prime }$ is the threshold used in CD-HIT's short word filter.

### The characteristics of the modified short word filter

As mentioned above, the short word filter used in our method enumerates every *k*th *k*-mer of cluster representatives and all *k*-mers of the query sequences. By contrast, CD-HIT's short word filter enumerates all the *k*-mers of the cluster representatives and query sequences. There are two main advantages of enumerating and registering every *k*th *k*-mer instead of all the *k*-mers. The first is that the size of the short word table in main memory is reduced to about 1/*k*. Second, the computation time necessary for searching cluster representatives in the short word table is also reduced because the number of entries decreases to about 1/*k*.

On the other hand, there are several possible demerits. Our short word filter uses a lower threshold *t *than that of CD-HIT; therefore, it is possible that the number of dissimilar sequence pairs may increase because of coincidental partial matches between sequences. Also, short word filtering fails to find a common *k*-mer between similar sequences if mismatches and gaps between a cluster representative and the query sequence appear at equal intervals.

However, the first demerit can be addressed by introducing a fast and more accurate filtering process after the short word filter and before the alignment process. Our method uses LCS filtering for this purpose. The second demerit occurs very rarely, because DNA sequences are not random sequences, and it can be considered as a rare case that mismatches and gaps in a sequence pair will appear at equal intervals, even when their sequence identity meets the threshold *s*.

## Results and discussion

### Performance evaluation

We implemented our clustering method in C++ and compared its efficiency and accuracy with those of CD-HIT. For this evaluation, we used two different types of datasets of short reads. One type comprised artificial datasets, including short reads, generated by the MetaSim software [14] from the *Bacillus amyloliquefaciens *genome sequence. The datasets included reads of fixed length. We used three different patterns of sequence lengths (100, 150, and 400 bases) and three different dataset sizes (one million, two million, and five million sequences). Thus, the total number of the datasets was nine. The other datasets comprised real sequencing data for metagenomic samples obtained by Roche's 454 and Illumina/Solexa sequencers. The 454 dataset includes 34,719 reads of lengths from 41 to 629 bases, and the Illumina dataset includes 6,822,944 reads with lengths from 60 to 75 bases.

We set the thresholds and parameters as follows: the threshold of sequence identity *s *was 0.9, the length of short word *k *was 9, the thresholds of the number of common *k*-mers *t *were 1 (for 100 bases and 150 bases) and 4 (for 400 bases). The setting of each parameter, especially sequence identity *s*, is highly dependent on the application and it should be determined during the research. Here, we used a value of *s *that has often been used in previous metagenomic analysis [10]. We ran the clustering programs on a workstation running SUSE Linux 10 with a single-core AMD Opteron processor (2.8 GHz) and 32 GB of memory.

Tables 1, 2, 3 show the computation times of the clustering processes for the artificial sequencing datasets. Performance ratios of LCS-HIT to CD-HIT are shown in parentheses.

**Table 1:** Computation time for each sequence length (1 million sequences)

	100 bases		150 bases		400 bases	
CD-HIT	41m40s		45m15s		1h4m29s	
LCS-HIT	7m10s	(5.8)	13m45s	(3.3)	31m22s	(2.1)

**Table 2:** Computation time for each sequence length (2 million sequences)

	100 bases		150 bases		400 bases	
CD-HIT	2h11m47s		2h17m56s		2h50m38s	
LCS-HIT	18m42s	(7.1)	31m41s	(4.4)	1h7m26s	(2.5)

**Table 3:** Computation time for each sequence length (5 million sequences)

	100 bases		150 bases		400 bases	
CD-HIT	11h17m22s		11h28m17s		14h57m56s	
LCS-HIT	2h11m09s	(5.2)	3h4m43s	(3.7)	6h42m23s	(2.2)

**Figure 4:**
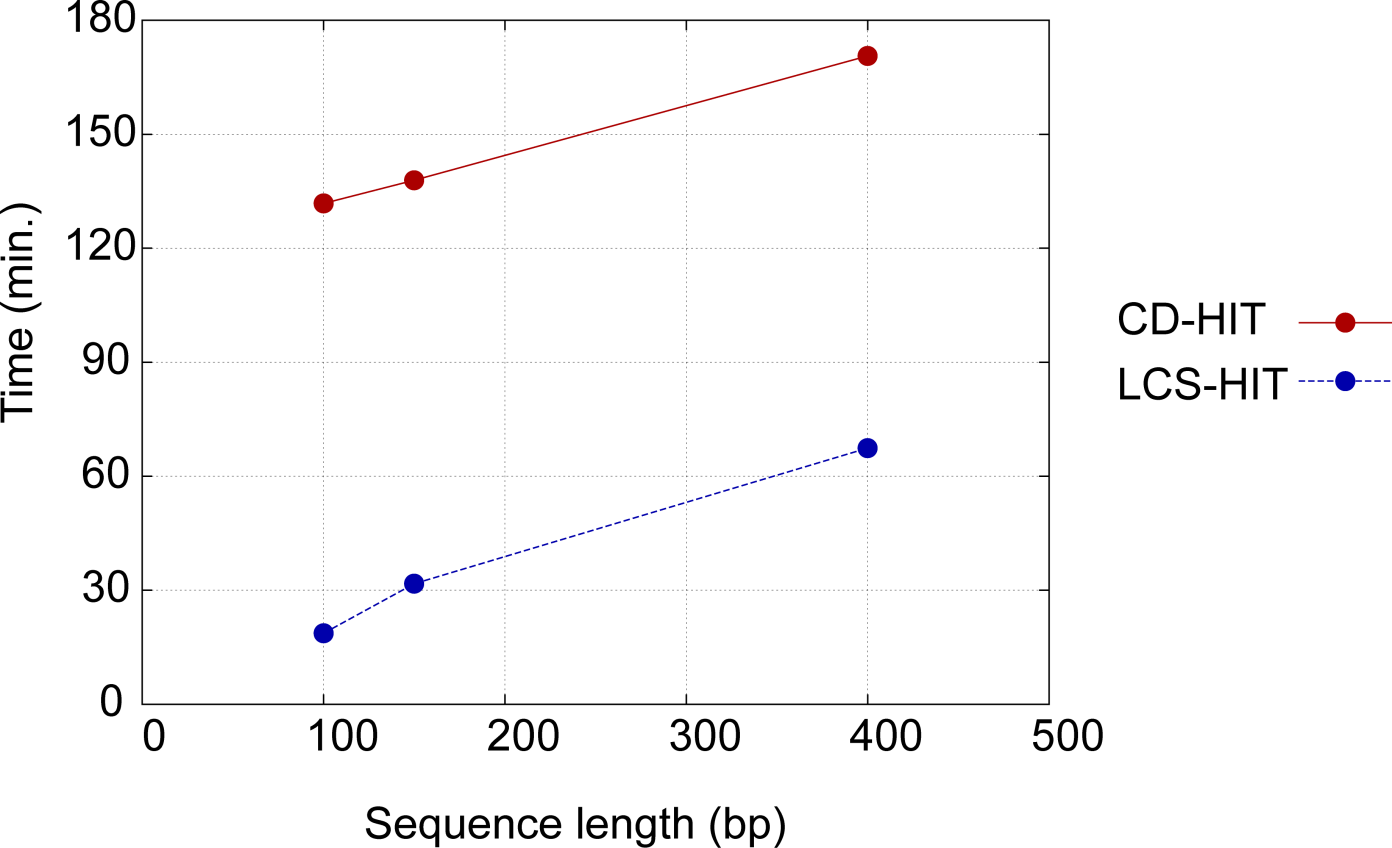
**Computation time (2 million sequences)**. The red line shows the computation time of LCS-HIT for each read length and blue line shows that of CD-HIT.

These results clearly show that LCS-HIT was faster in all cases. For the dataset with two million DNA sequences, our method was approximately 7.1, 4.4, and 2.5 times faster than CD-HIT for 100, 150, and 400 bases, respectively. The speed enhancement was large for shorter sequences, whereas longer sequence lengths tended to obtain less improvement with the proposed algorithm. One of the reasons for this is that the longer are the sequences, the greater is the number of cases where sequence pairs with a sequence identity less than the threshold have an $LLCS\left(X,Y\right)/n)$ value larger than the threshold. This is because there is more opportunity to make an alignment with sufficient matches using many gaps in a longer sequence pair. Thus, both our method and CD-HIT suffer the same increases in computational time for increases in sequence length (Figure 4). This problem might be solved by considering gaps in LCS alignments and compensating the LCS score.

Table 4 shows the number of clusters generated by each method. Although there is no large difference between the clustering results, CD-HIT sometimes did not correctly cluster similar sequence pairs that LCS-HIT could correctly assign. The short word filter in CD-HIT is too strict, and even similar sequence pairs sometimes fail to pass through. However, our modified short word filter is relatively loose, allowing more non-similar sequence pairs to pass. Nevertheless, almost all similar sequence pairs pass through our filter.

**Table 4:** Number of clusters (2 million sequences)

	100 bases	150 bases	400 bases
CD-HIT	1,242,054	1,015,466	493,384
LCS-HIT	1,185,704	970,419	480,201

We also applied our method to real sequencing datasets, Roche's 454 and Illumina/Solexa reads, as shown in Table 5. Our method outperformed CD-HIT for both datasets, but did especially well for Illumina/Solexa, probably because of the shorter reads in the Illumina/Solexa data.

**Table 5:** Computation time for real sequencing datasets

	454		Illumina/Solexa	
CD-HIT	2m47s		27h2m5s	
LCS-HIT	44s	(3.8)	3h44m31s	(7.4)

## Discussion

LCS-HIT uses, at most, 1.6 times more memory than CD-HIT; it was about 5 GB for five million 400-base sequences. Our method only registers every *k*th *k*-mer in the short word table; therefore, the memory required for our short word table is less than that required by CD-HIT. However, the CD-HIT program sets an upper boundary for memory usage by the short word table (800 MB by default), and can run in this limited memory space. Therefore, overall, CD-HIT uses less memory than LCS-HIT, even though our method has a smaller short word table. There is still room for improvement in the memory usage of LCS-HIT, and thus it cannot be concluded that our method is inferior in this respect at this point.

In this work, we used a bit-parallel algorithm for calculating the length of LCS. However, much faster implementations of the LCS calculation have already been proposed. For instance, Chen et al. proposed an Message Passing Interface (MPI)-based fast parallel algorithm for finding the longest common sequence [15] and Yang et al. proposed an efficient parallel algorithm on GPUs [16]. Thus, clustering may be accelerated using these methods. However, from the profiling of the program, the LCS calculation consumed less than 5% of total computation time, thanks to the bit-parallel algorithm. Therefore, it is difficult to increase the speed of the whole clustering process by accelerating only the LCS calculation.

## Conclusions

We developed the LCS-HIT fast clustering algorithm for DNA sequence data, which employs a new filtering scheme based on the longest common subsequence (LCS). This filtering scheme allows accurate pruning of dissimilar sequence pairs that are not discarded by short word filtering alone. Thus, it accelerates the clustering process as a whole.

The LCS filter is also effective as a second filter when relaxing the filtering criterion of the short word filter to reduce computation time. For two million DNA sequences, LCS-HIT was about 7.1, 4.4 and 2.5 times faster than CD-HIT for 100, 150, and 400 bases, respectively. LCS-HIT will enable clustering of huge DNA datasets that cannot be handled with conventional sequence clustering tools in a reasonable amount of time. Moreover, the filtering technique itself is independent from the CD-HIT algorithm. Thus, this technique can be applied to similar clustering algorithms.

## Competing interests

The authors declare that they have no competing interests.

## Authors' contributions

YN carried out the study, implemented the method, analysed the results and wrote the manuscript. TI designed the study, analysed the results and wrote the manuscript. YA conceived of the study and wrote the manuscript. All authors read and approved the final manuscript.

## Declarations

The publication of this research is funded by the HPCI STRATEGIC PROGRAM in Computational Life Science and Application in Drug Discovery and Medical Development by MEXT of Japan. This research was supported in part by the Cancer Research Development Funding by the National Cancer Center of Japan.

This article has been published as part of *BMC Bioinformatics *Volume 14 Supplement 8, 2013: Proceedings of the 2012 International Conference on Intelligent Computing (ICIC 2012). The full contents of the supplement are available online at http://www.biomedcentral.com/bmcbioinformatics/supplements/14/S8.
